# Dynamical Signatures of Multifunnel Energy Landscapes

**DOI:** 10.1021/acs.jpclett.2c01258

**Published:** 2022-07-08

**Authors:** David J Wales

**Affiliations:** Department of Chemistry, University of Cambridge, Lensfield Road, Cambridge CB2 1EW, U.K.

## Abstract

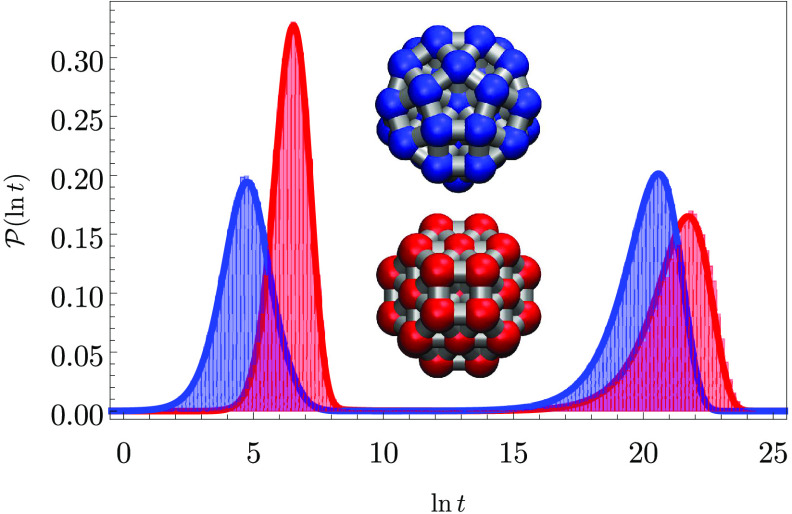

Multifunctional systems, such as molecular switches, exhibit multifunnel energy landscapes associated with the alternative functional states. In this contribution the multifunnel organization is decoded from dynamical signatures in the first passage time distribution between reactants and products. Characteristic relaxation rates are revealed by analyzing the kinetics as a function of the observation time scale, which scans the underlying distribution. Extracting the corresponding dynamical signatures provides direct insight into the organization of the molecular energy landscape, which will facilitate a rational design of target functionality. Examples are illustrated for multifunnel landscapes in biomolecular systems and an atomic cluster.

The relaxation times measured in an experiment are determined by the structure of the underlying energy landscape, which encodes the pathways between products and reactants, and the associated barriers and rates. To interpret the corresponding properties, we need to know what is actually being measured and the time scale of the observation. This time scale determines which transformations are feasible^1^ and which states define the free energy minima that are averaged over in local equilibrium.^2−5^ Distinguishing different product and reactant states on different time scales has direct consequences for observations, ranging from resolution of tunnelling splittings in spectroscopy to thermodynamic measurements subject to broken ergodicity.

Interconversion rates report on the mean first passage time (MFPT) between reactant and product states. In this contribution we demonstrate how an analysis of the first passage time (FPT) distribution provides access to a wealth of additional information, including direct information on the organization of the underlying energy landscape. In particular, it enables us to resolve the signatures corresponding to distinct funnels, associated with local free energy minima^6,7^ and kinetically convergent pathways.^8^

Single funnel landscapes correspond to good structure-seeking systems, such as magic number clusters and biomolecules that have evolved to fulfill a particular function. Multifunnel landscapes provide the opportunity to encode multiple functions, with the potential to act as molecular switches or bind alternative partners that stabilize particular structural motifs.^9−11^ The existence of multiple relaxation times could also be significant in terms of functionality. The dynamical signatures of landscapes capable of encoding multifunctional properties are therefore of great interest. Avoiding such kinetic traps is also a key principle in the design of efficient self-assembly.^12^

Double funnel landscapes have previously been characterized for atomic clusters with competing low-energy morphologies.^13−16^ These clusters exhibit two characteristic slow relaxation time scales, one for direct relaxation to the global minimum and the slowest for paths that are diverted first to the competing funnel and must then escape from this kinetic trap. The present results show how the dynamical signatures of multiple funnels are connected to the organization of the landscape and how the observation time scale determines the outcome of corresponding measurements. Competing funnels that correspond to kinetic traps produce distinct peaks in the first passage time distribution on a logarithmic time scale,^17^ consistent with long time tails associated with protein folding,^18^ and multiexponential kinetics.^19−22^ Here we address the underlying kinetic transition network directly, to analyze how characteristics of the landscape, especially kinetic traps and the corresponding barriers, translate into features of the first passage time distribution. The theory applies to any network and any observation time scale where the kinetics are described by a linear master equation. This approach is complementary to schemes that focus on observables for single-molecule kinetics,^23^ where first passage time information has previously been exploited to make mechanistic inferences^24^ and detect intermediate states.^25^ These previous applications to colloidal dynamics, folding kinetics, and transport through a pore provide some indication of the potential insight that might be achieved in future work. The present contribution reveals how multiple kinetic traps can be detected and visualized directly from an analysis of the first passage time distribution. It also demonstrates that corresponding features arise in the mean first passage time as the observation time scale of an experiment changes.

## Analysis of the First Passage Time Distribution

We consider the first passage time distribution between product and reactant states, denoted  and , for a kinetic transition network (KTN).^26−28^ We assume that the network has already been created using some rare events methodology, which could be based on geometry optimization and coarse-graining into minima and transition states of the underlying energy landscape via discrete path sampling,^29,30^ or methods based on explicit dynamics.^31−35^ Having obtained a KTN, with nodes corresponding to local potential or free energy minima, and edges associated with forward and backward rates, the kinetics are extracted from a master equation formulation, as detailed in the Methods.

In the initial tests, the FPT distribution was calculated using both an analytical formulation and kinetic Monte Carlo (kMC) methods to validate the results and test convergence. The analytical formulation follows from a direct analysis of the rate equations via eigendecomposition.^17,37^ As shown in the Methods, the first passage time distribution *p*(*t*) and the corresponding probability distribution for *y* = ln *t*, , can be written as
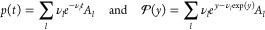
1where −ν_*l*_ are the eigenvalues of the matrix defining the master equation dynamics, with ν_*l*_ > 0, and the *A*_*l*_ are amplitudes, which depend on the eigenvectors of the matrix and the initial condition defined by the reactants. Eigendecomposition results from routines DSYEVR and DGEEV were checked for consistency, as in previous work.^17,37^ Real negative eigenvalues are assured because the absorbing Markov chain derives from a Markov process that is reversible. The distribution for ln *t* is particularly useful because it separates the distinct time scales clearly, as illustrated below.

To examine the dependence of the MFPT on the observation time scale, we can integrate *tp*(*t*) up to a cutoff time *t*_obs_, giving
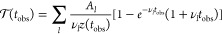
2where *z*(*t*_obs_) is the normalization for the restricted distribution. Hence we might interpret this quantity in terms of transformations observed in an ensemble of molecules, where the experiment measures a signal corresponding to appearance of product or disappearance of reactant. We are therefore considering “feasible” kinetic processes, analogous to feasible operations that determine the appropriate molecular symmetry group and tunnelling splittings for nonrigid molecules.^1^

**Figure 1:**
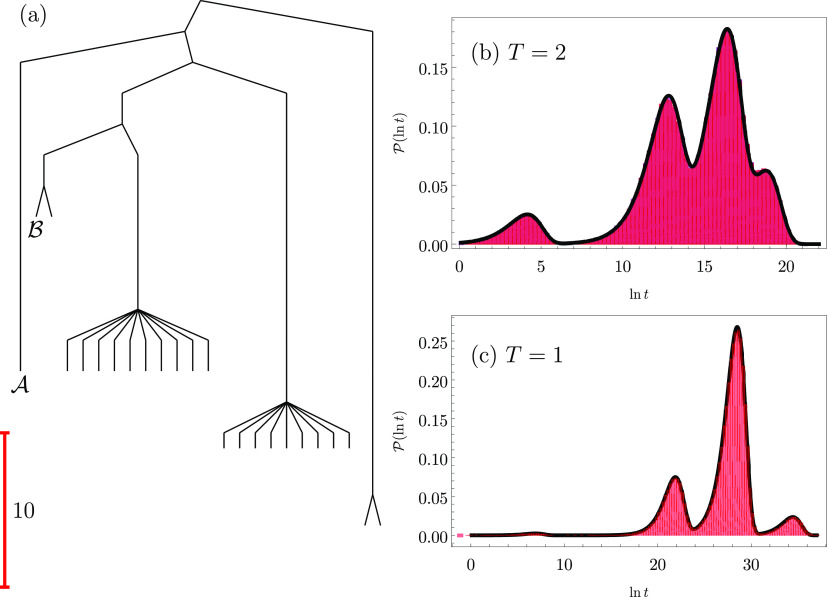
(a) Disconnectivity graph^13,36^ for a model energy landscape that features multiple kinetic traps. The scale bar corresponds to 10 energy units, and the product and reactant states  and  are marked. In this representation, the vertical scale corresponds to potential energy, and branches terminate at the energies of all the local minima, arranged on the horizontal axis to best illustrate the organization of the landscape. The branches merge when the energy exceeds the highest transition state on the lowest energy path between the corresponding minima, thus providing a faithful account of the barriers.^36^ (b, c) Probability distributions of ln *t* at temperatures *T* = 2 and *T* = 1; the peak positions for increasing time correspond to the four traps with increasing depth, which correlate directly with the arrangement in the graph from left to right. For *T* = 2 in (b) two histograms are superimposed for kMC and leapfrog kMC results in purple and red, respectively. The solid lines correspond to the analytical solution obtained by eigendecomposition in eq 1. All the kMC results are well converged for 200 000 kMC runs.

When evaluated numerically the analytical results in eq 1 eventually suffer from loss of precision as the temperature decreases. Standard rejection-free kMC^38−40^ schemes also enable the FPT distribution to be extracted, but they become unfeasible at low temperatures because the number of steps increases rapidly as the temperature decreases. However, a formulation that samples the distribution of transition times for second-neighbor jumps (leapfrog kMC, Methods) is effective for the model landscapes used to test all the methodology. Results are first presented for one such model, which is representative of the test cases considered. Quantitative agreement was obtained between eigendecomposition, conventional kMC, and leapfrog kMC in the temperature ranges where alternative approaches were numerically tractable. The model is defined by a kinetic transition network,^26−28^ which includes all the information required to calculate the partition functions for the minima and transition states, using equilibrium statistical mechanics and unimolecular rate theory to extract thermodynamic properties and the rate constants between connected minima, as reviewed elsewhere.^10,41^ The corresponding landscape is designed to feature kinetic traps and an associated range of time scales, with local minima separated by low barriers in four branches of the disconnectivity graph shown in Figure 1. These minima exhibit multiple recrossings in kMC trajectories, highlighting the “flickering” problem in kMC simulations^42,43^ and increasing the escape time from the traps.

**Figure 2:**
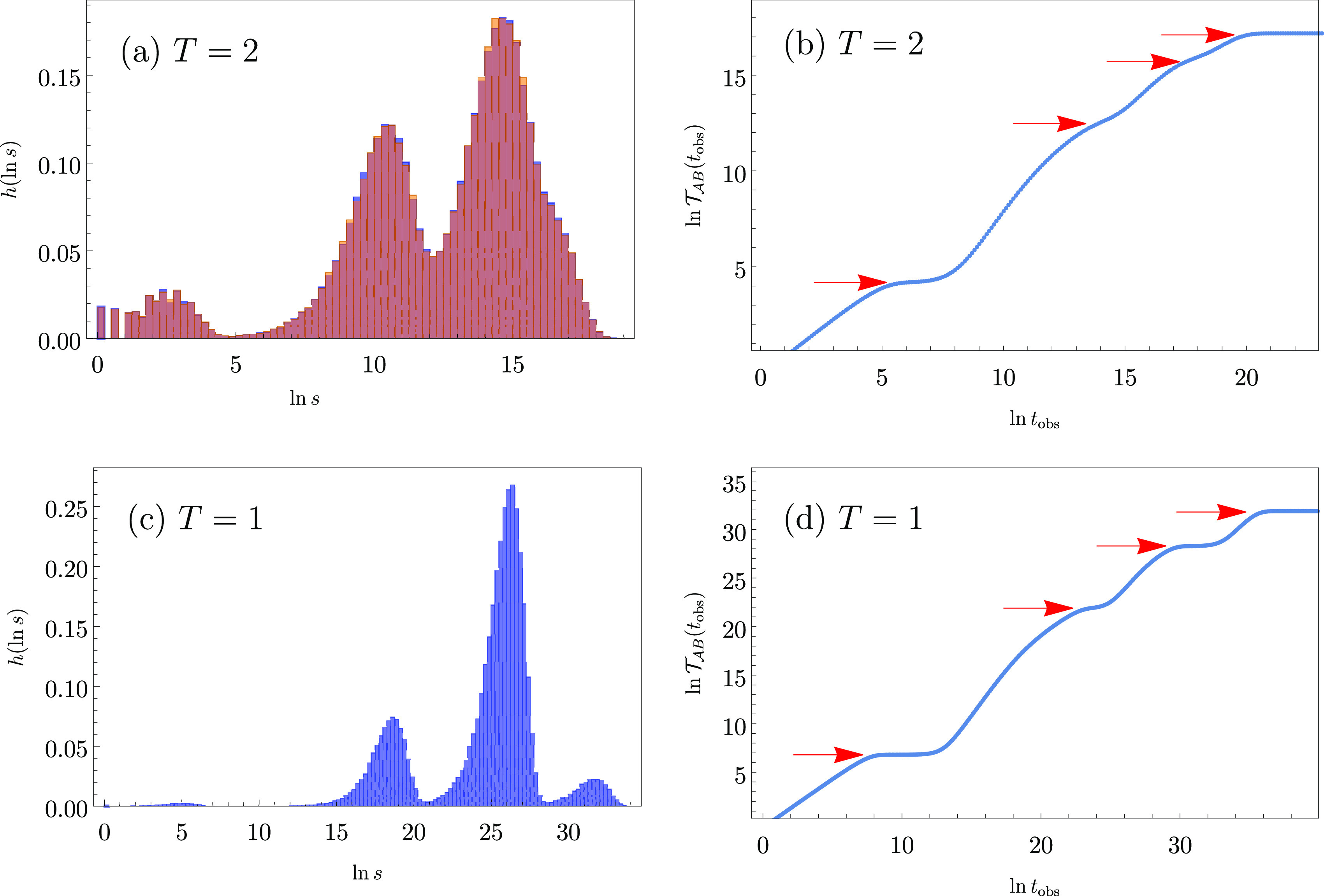
Probability distributions of ln *s* at temperatures (a) *T* = 2 and (c) *T* = 1 for the model landscape shown in Figure 1. For *T* = 2 two histograms are superimposed for kMC and leapfrog kMC results in orange and blue, respectively. (b, d) Associated MFPT  as a function of the observation time scale cutoff *t*_obs_, calculated from the analytical eigendecomposition formulation in eq 2. The red arrows mark the position of steps in  predicted from eq 1, which correspond closely to the peaks in  in Figure 1.

Figures 1 and 2 illustrate representative results at temperatures *T* = 2 and *T* = 1, below which the eigendecomposition becomes numerically unstable. Temperature is measured in the same units as the potential energy corresponding to Figure 1a. Four peaks are evident in the probability distributions  and *h*( ln *s*) for the first passage time *t* and the corresponding number of kMC steps *s*, which is also known as the dynamical activity.^44,45^ Hence these distributions report directly on the kinetic traps and the corresponding barriers that must be overcome to escape them. At the lower temperature the peaks are shifted to significantly higher values of *t* and *s*, especially for the three slower features, which correspond to escape from the three deeper traps in the landscape (Figure 1). The peaks are also more clearly separated at *T* = 1. Hence there is a direct correspondence between the organization of the landscape and the probability distributions for the first passage time and the dynamical activity. We see quantitative agreement between the eigendecomposition and kMC results (where accessible) for these examples, which are representative of all the runs conducted at different temperatures for various model landscapes. The four peaks correspond to trajectories that reach  via the four distinct branches on the landscape, starting from .

The effect of cutting off the average for the mean first passage time at a maximum observation time *t*_obs_ can be explored using eq 2. Selected results are shown in Figure 2. We see that  exhibits four distinct steps corresponding to escape from the four kinetic traps.  converges to the usual mean first passage time, , in the limit of long observation time scales, where
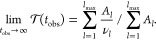
3The four arrows in Figure 2b,d correspond to performing the sums in eq 3 up to *l*_max_, *l*_max_ – 1, *l*_max_ – 2, and *l*_max_ – 3, where  corresponds to the slowest relaxation. In the temperature range where the analytical results are reliable, the value obtained for  agrees precisely with graph transformation,^37,46−49^ which provides a numerically stable deterministic method for calculating this quantity.

Equation 2 shows that distinct features are expected in  if the slow relaxation time scales defined by the values of 1/ν_*l*_ are well-separated. This situation corresponds to kinetics where accurate fits might be obtained as a combination of exponential decay processes.^19,21,22^ In contrast, for more complex systems, such as glasses, we would not expect to resolve such features.

## Results for Molecular Multifunnel Landscapes

We now consider applications to multifunnel landscapes for an atomic cluster, a designed bistable peptide, and an RNA switch. Full details of how these databases were created are available in previous work;^16,50,51^ here we show that an analysis of the first passage time distribution and the observation time scale provides new insight into the dynamics, with a direct connection to the underlying energy landscape.

**Figure 3:**
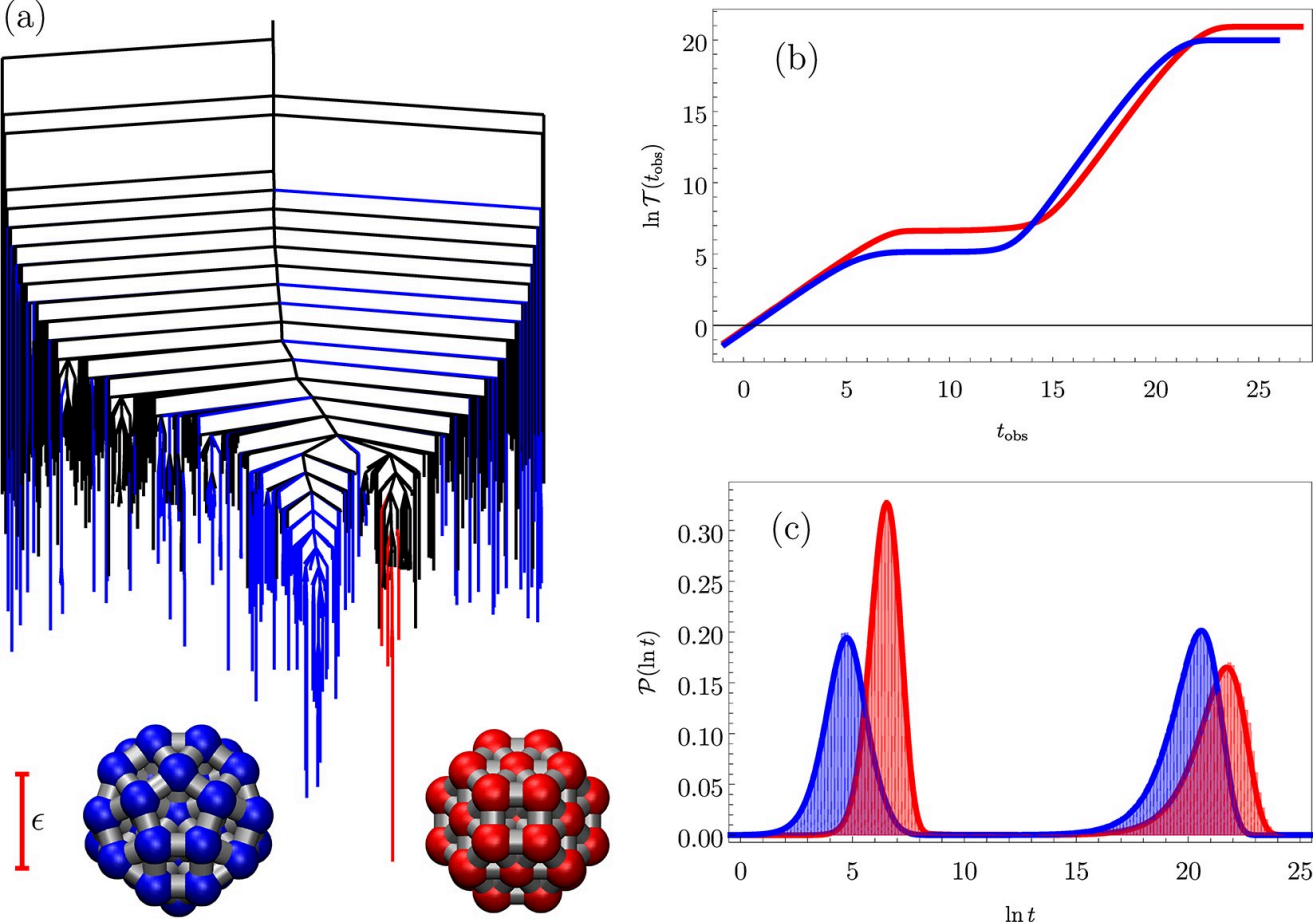
(a) Disconnectivity graph^13,36^ for an LJ_38_ database containing 4000 minima with the branches for  (icosahedral) and  (cuboctahedral) minima colored blue and red, respectively. The global minimum () and second-lowest minimum () are illustrated; ϵ is the pair well depth. (right) FPT diagnostics for relaxation to  or  with the initial population at *t* = 0 localized in a high energy local minimum. These results correspond to eigendecomposition at *k*_b_*T*/ϵ = 1.25. (b)  exhibits two clear steps corresponding to (c) bimodal probability distributions in  for relaxation to either  or . The shorter time scale is for direct relaxation in each case, and the long time scale results from trajectories that are diverted into the competing structure and must then escape from this kinetic trap.

The 38-atom cluster LJ_38_ bound by the Lennard-Jones potential provides a well-understood example of a double-funnel molecule,^13,15,16^ with low-lying structures based on close-packed  and icosahedral  structures (Figure 3). The same database and product/reactant definitions were used here as in previous work.^17^ Two time scales are clearly visible in the probability distributions for ln *t* and  for relaxation to either  or  when the initial occupation probability is localized in a high-lying minimum. These results are for a reduced temperature of *k*_b_*T*/ϵ = 1.25, just above the equilibrium temperature between the two morphologies, at the limit where eigendecomposition is numerically stable. For relaxation to , the  region acts as a kinetic trap, and vice versa, producing the peaks in  around ln *t* = 22 and ln *t* = 20. These longer time-scale features correspond to switching between the two competing morphologies. The peaks around ln *t* = 7 and ln *t* = 5 are for direct relaxation to  and , respectively.

The precise features observed in  depend on the initial distribution. For all three examples considered here the reactants were selected to highlight distinct relaxation time scales. Different experimental setups could probe the dependence of relaxation times on the preparation of the initial conditions, which could provide further insight into the organization of the landscape.

**Figure 4:**
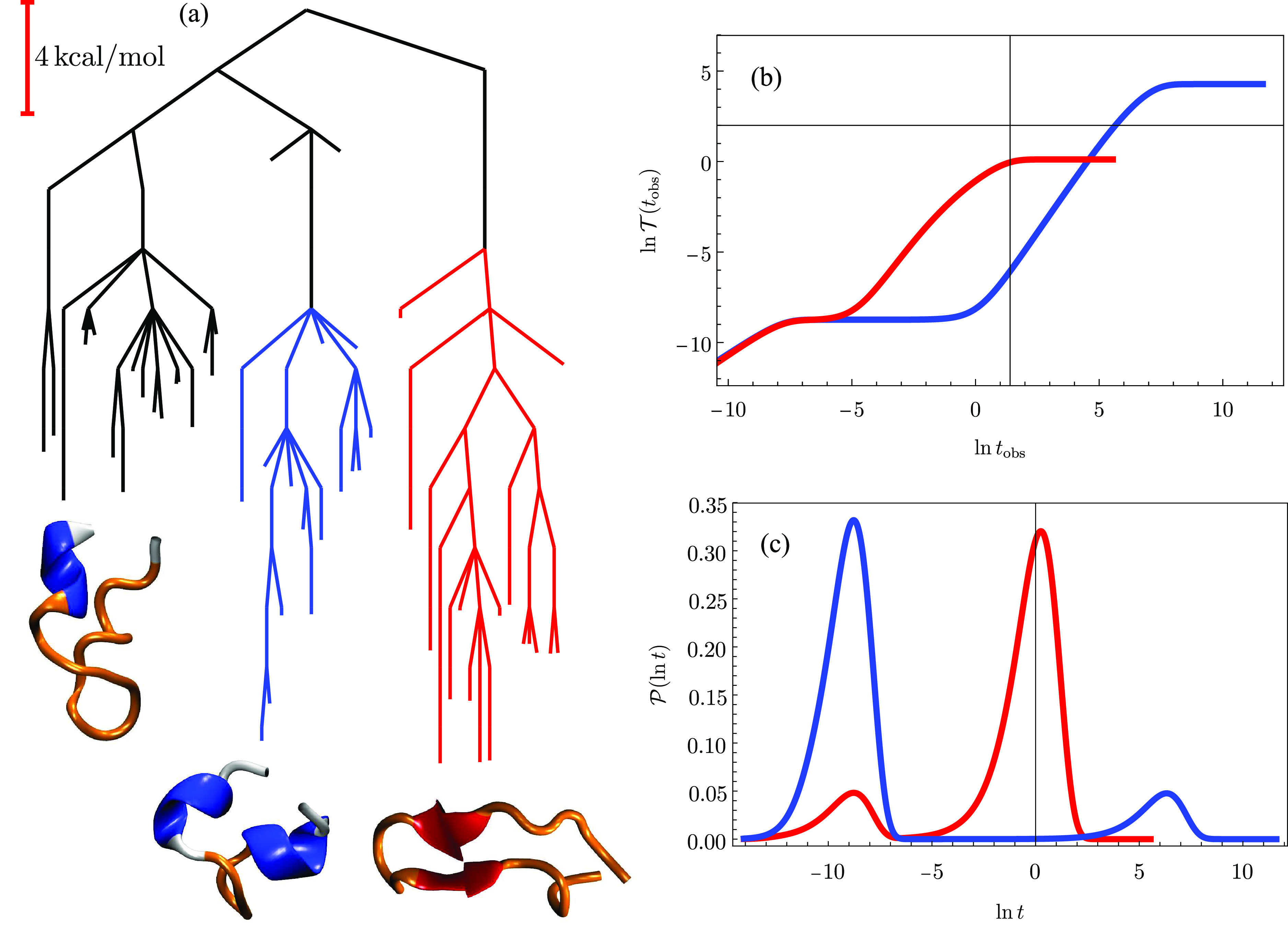
(a) Disconnectivity graph^13,36^ for a subset of the database^51^ for the DP5 peptide^52^ containing 54 minima. The β-hairpin (red) and the lowest energy minimum in the α-helical (blue) basin are shown near the corresponding branches in the graph. (right) FPT diagnostics calculated at 300 K from eigendecomposition with the initial population at *t* = 0 localized in the high-energy local minimum illustrated. (b)  exhibits two clear steps corresponding to (c) bimodal probability distributions  for relaxation to both β and α secondary structures. Both *t*_obs_ and *t* are in seconds.

Figure 4 shows results for the 18-residue peptide DP5, which was designed to exhibit competing α-helical and β-hairpin structures.^52^ The interconversion mechanism is of interest for the insight it may provide into pathways to amyloid formation. A subset of the database was selected that supports low-lying hairpin and helical morphologies and the key interconversion pathway between them. Relaxation to both these structures from a higher energy minimum at 300 K reveals two peaks in  corresponding to direct and indirect relaxation via the α and β regions.  exhibits two steps in each case.

**Figure 5:**
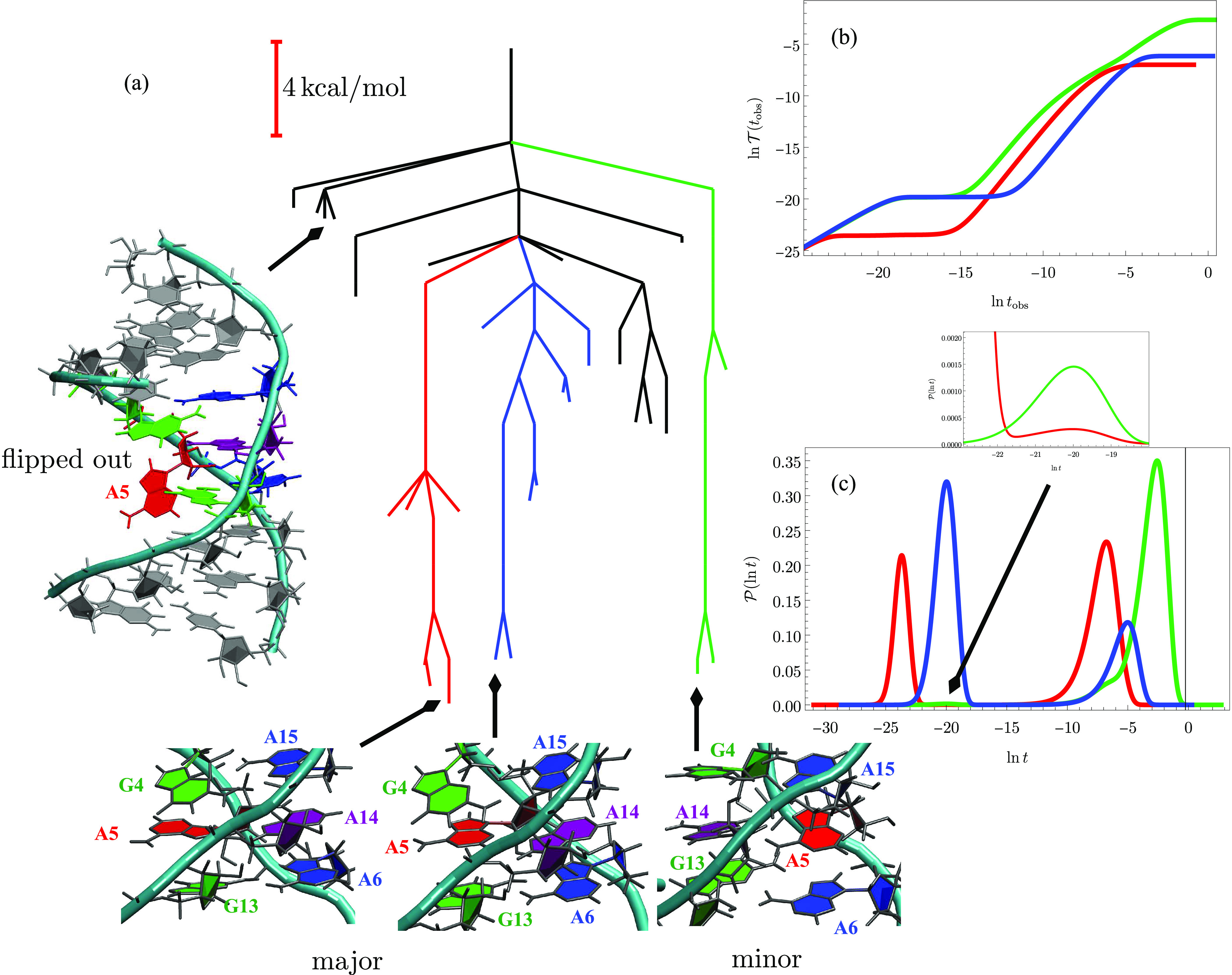
(a) Disconnectivity graph^13,36^ for a subset of the database^50^ for a nine base pair RNA duplex^53^ containing 29 minima. The FPT distributions correspond to relaxation from the minimum with adenine A5 flipped out, shown in full at the top left of the graph, to three target minima. Two of these structures correspond to the major form (red and blue) with A14 stacked between A6 and A15, and the third target is the minor form (green), where A5 is stacked between A6 and A15. The corresponding structures are illustrated for magnifications of the central base region to highlight the alternative base stacking. The two distinct components of the major funnel, highlighted in red and blue, differ by a 180° rotation of the A5 purine ring about the bond to the ribose ring. (right) FPT diagnostics calculated at 300 K from eigendecomposition; all times are in seconds. (b)  exhibits two clear steps for relaxation to the two major form minima corresponding to (c) bimodal probability distributions .  for the minor form has an additional feature around , which produces a shoulder in the  distribution. (inset) Magnification of the region around ln *t*/s = −20, revealing additional small peaks in .

Figure 5 shows the results for a nine base pair RNA duplex that features a noncanonical adenine–adenine base pair. NMR results show that this system exhibits switching between major and minor forms, and previous simulations indicate that the dominant kinetic pathways feature stacked intermediates.^50,54^ Another funnel containing structures with the A5 base flipped out was found to be favorable at higher temperature, where the balance between enthalpy and entropy shifts.^50^ One of these minima with A5 flipped out was selected as the starting point, and the figure shows results for relaxation to three low-lying minima in the major and minor forms at 300 K. Three distinct time scales are discernible for the minor form target, with steps in  translating directly into peaks for the  distribution. The peaks corresponding to the two slower processes overlap, producing a shoulder in the distribution around ln *t*/seconds = −6.

## Discussion

The mean first passage time between reactants and products is the usual focus of attention for an analysis of rates in systems that exhibit conventional single-exponential kinetics. However, for systems that exhibit multiple relaxation time scales the first passage time distribution encodes information that reports directly on the structure of the underlying energy landscape. Such behavior is expected for designed or evolved molecules that can act as switches, where the equilibrium between competing structures may be shifted by external factors. In particular, the ability of experiments to probe a wider range of time scales at higher resolution has the potential to provide new insight into such phenomena and probe the multifunnel/multifunctional hypothesis.^9^ Evolved biomolecules that perform different cellular functions provide a testing ground;^10,11^ for example, intrinsically disordered proteins may correspond to intrinsically multifunctional systems, where function is encoded in a multifunnel landscape.

To connect the features of the first passage time distribution with experiment we must take account of what is actually being measured; the question of what to calculate logically precedes the problems associated with how to do the calculation. The first issue is the definition of reactant and product states, which may combine structures with analogous experimental signatures that are separated by insurmountable barriers. These structures must be lumped together in the analysis of kinetics; otherwise, additional slow relaxation processes will interfere with the interpretation of the data. The accessible experimental time scale is also key. If our calculations can probe processes corresponding to kinetic traps that are not observable, then including these transformations in the analysis will produce calculated rates that are too slow. We should therefore focus on feasible processes, which produce observable experimental signatures in the same way that the molecular symmetry group is defined for nonrigid molecules in terms of observable tunnelling splittings.^1^ The results also suggest that caution may be required when rates are calculated by inverting mean first passage times, which may be a particular issue for multiscale or coarse-grained models.

The feasible molecular rearrangements also determine the appropriate free energy minima,^5^ since the concept of equilibrium is determined by the observation time scale^2^ and, hence, the interplay with kinetics. In this contribution, these issues have been addressed directly, by analyzing the first passage time. The time scales associated with escape from kinetic traps in the energy landscape can produce clear signatures in the first passage time distribution and in the mean first passage time calculated as a function of the observation time scale. These features are resolvable for processes that correspond to well-separated time scales and become more distinct at a lower temperature. The effective time scale could also be tuned by changing experimental conditions, such as temperature or the strength of an applied field. The techniques developed here will enable detailed information about the organization of the energy landscape to be extracted and, hence, facilitate the design of multifunctional materials.

## Methods

### First Passage Time Distributions from Eigendecomposition

The first passage time probability distribution *p*(*t*) can be obtained by considering the products as an absorbing state, since the dynamics are unchanged up to the point of absorption.^35,37^ Considering  for specificity, and denoting the other intervening states as , the appropriate master equation for the occupation probabilities *P*_α_(*t*) for states in  is
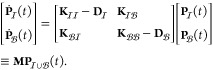
4

Here  is the matrix containing the individual rate constants between connected states, with dimension  is a diagonal matrix of dimension  with elements corresponding to the total escape rates from each state in , so that . When the  states are absorbing the escape rates from  are zero, so , but transitions to  appear in  and .

The formal solution to eq 4 is . Equating the probability flux out of  with the probability that the first passage time for  lies between *t* and *t* + d*t* implies that
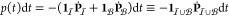
5for initial distribution , where  is a row vector of ones with dimension , etc. Hence
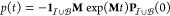
6which we now write in terms of the eigendecomposition of **M**
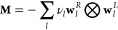
7where ⊗ is the is the diadic (outer) product, the eigenvalues −ν_*l*_ < 0 are negative for a connected network, and **w**_*l*_^L^ and **w**_*l*_^R^ are the left (row) and right (column) eigenvectors of **M**, with **w**_*l*_^L^**w**_*q*_^R^ = δ_*lq*_. The probability distribution for the first passage time is therefore
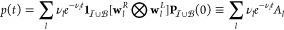
8The moments of this distribution are the expectation values of *t*^*n*^
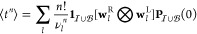
9with MFPT  and variance  corresponding to ⟨*t*⟩ and ⟨*t*^2^⟩, respectively.

In the present analysis we used two extensions of these results. To highlight the dynamical signature of a multifunnel landscape we need the probability distribution of *y* = ln *t*, denoted , with
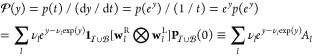
10We can also calculate the moments corresponding to experiments with a maximum observation time scale *t*_obs_ by integrating up to this limit instead of infinity to obtain
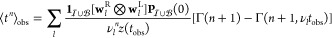
11with normalization
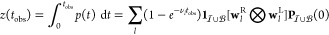
12In particular
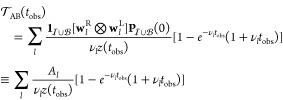
13 is the MFPT averaged over events that occur on the accessible observation time scale.

### First Passage Time Distributions from Leapfrog Kinetic Monte Carlo

The analytical formulations break down at low temperatures because of numerical precision problems.^17,48^ The low-temperature regime is often the focus of interest, where the key transformations correspond to rare events. To access the FPT distribution we have previously employed kinetic path sampling (kPS),^42,55^ which uses the graph transformation (GT) approach.^37,46−49^ kPS analyses escape from subnetworks in a coarse-grained representation of the original network and samples the master equation exactly.^42,55^ The graph transformation component enables kPS to be used in the metastable regime, so long as the subnetworks describe the metastable sets of nodes appropriately.^43^ This framework can provide first passage time distributions at temperatures where conventional kMC procedures^38−40^ are unfeasible due to flickering between nodes and excessively long path lengths.^42,43^ Graph transformation^37,46−49^ (GT) can be used to calculate the mean first passage time reliably at low temperature and provides a reference value for comparison with averages over the FPT distribution.

The FPT distributions for the model landscapes considered as test cases in the present work can be obtained efficiently using an extension of the leapfrog kMC scheme, introduced in earlier work to mitigate the flickering problem.^29,56^ In this method we employ renormalized branching probabilities and waiting times for the current minimum in a kMC simulation, to allow steps to second neighbors or directly connected absorbing states, if there are any.

Using the expectation value for the waiting time over all the implicit transitions conserves the overall MFPT between reactants and products, as in the GT approach. For the current state *i*, with neighbors , we consider steps to any neighbor of *i* in the absorbing set  and to all second neighbors of *i* that are directly connected to , excluding *i* itself. These events can be combined with any number of *i* ↔ *j* recrossings in any order. The relative probability of *n* total recrossings with *n*_*j*_ for neighbor *j* and  is
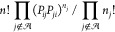
14which is a term in the multinomial expansion . Here *P*_*ij*_ is the branching probability of moving to connected minimum *i* from minimum *j*. The probability of *n* total recrossings is
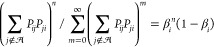
15with . The probability of stepping to any second neighbor *k* ≠ *i* with any number of recrossings is , and the probability of stepping to any neighbor of *i* in  is , which together sum to one. The path probabilities are conserved if we renormalize  for *k* ≠ *i* and *P*_*ji*_^lf^=*P*_*ji*_/(1−β_*i*_) for , and the MFPT to products is conserved by setting ,^56^ where τ_*i*_ is the expected waiting time for a transition out of minimum *i*.

### Sampling Distributions with Leapfrog Kinetic Monte Carlo

To calculate the full FPT in the present analysis, rather than just the mean value that was the focus of interest in the original development, we sample the number of kMC steps and associated waiting time for each leapfrog move from the appropriate probability distribution. Repeated kMC trajectories then sample the FPT distribution, which generally appeared well-converged for 100 000 repeats using different random number sequences. The results presented are based on sets of 200 000 leapfrog kMC runs, which only required a few seconds of computer time in these tests. In contrast, conventional kMC simulations and eigendecomposition cannot access the FPT at lower temperature. Some of the leapfrog trajectories considered in the results correspond to over 10^15^ conventional kMC steps.

To sample the number of recrossings, we note that the probability of *N* recrossings or fewer is (1 − β_*i*_)∑_*n*=0_^*N*^ β_*i*_^*n*^ = 1−β_*i*_^*N*+1^. Generating a random number *x* from a uniform distribution in the range (0, 1] and solving *x* = 1−β_*i*_^*N*+1^ produces *N* = ln(1 – *x*)/ ln β_*i*_ – 1 as the sampled number of recrossings, each of which involves two kMC steps. Sampling the associated waiting time is more involved, because the waiting time associated with each recrossing depends on the neighboring node *j*.

The recrossing problem corresponds to sampling *m* random variables, which may be collected in a column vector (*X*_1_,*X*_2_,···,*X*_*m*_)^*T*^, distributed according to a multinomial distribution with terms , where  and . For transitions out of minimum *i* this formulation corresponds to probabilities . For large *N* the multinomial distribution can be represented by a multivariate normal distribution, which can be sampled^57^ by generating *m* – 1 independent random variables identically distributed as , with
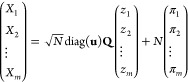
16where **u** is the vector with components , *z*_α_ is a random normal variable distributed as , and **Q** is an orthogonal matrix with final column **u**. diag(**u**) is the diagonal matrix with elements *u*_α_. Choosing **Q** = **I**_*m*_ – 2**v** ⊗ **v**^*T*^ can be shown to provide an appropriate formulation,^57^ for .

The multinormal sampling provides an approximate set of *n*_*j*_ values and associated waiting times *n*_*j*_(τ_*i*_ + τ_*j*_) for each leapfrog transition, which is accurate when  is large. Multinormal sampling was therefore applied for minima with β_*i*_ > 0.95. Alternatively, good agreement with eigendecomposition and standard kMC can also be achieved using the mean waiting time for a single recrossing step τ_*i*_^lf^, defined above, multiplied by the number of recrossings sampled from the appropriate distribution. The contribution of the exit steps and waiting times to a second neighbor or adjacent absorbing state are added to the recrossing contribution. For these exit steps the waiting time was sampled from a Poisson escape distribution using −τ_α_ ln *x* for a random number *x* drawn from the uniform distribution (0, 1] for each minimum α, as for the standard kMC runs. Nodes with only a single possible recrossing were treated separately, since we only need to sample the total number of recrossings for one edge of the network in that case, and the multinormal distribution is not required.

Leapfrog moves were used when β_*i*_ exceeded a given threshold; the results are not sensitive to this threshold, and all the values tested in the range from 0.1 to 0.9 worked very well. To avoid loss of precision when β_*i*_ approaches 1 we use the identity , as in previous work,^56^ where *j* and *k* run over first neighbors of *i* and first neighbors of *j*, respectively, excluding return to *i*.

I am grateful to a referee for noting the existence of an earlier alternative leapfrog KMC scheme,^58,59^ which could also have been used to check the eigendecomposition calculations. For more complex landscapes the kPS formulation would probably be needed,^42,55^ and as a further check kPS simulations were run for the model landscape in Figure 1 using the DISCOTRESS package.^60^ The FPT distributions obtained from these independent runs agree very well with the results obtained using eigendecomposition and the leapfrog scheme described above.
